# Transduction of ovarian cancer cells: a recombinant adeno-associated viral vector compared to an adenoviral vector

**DOI:** 10.1054/bjoc.2001.2082

**Published:** 2001-11

**Authors:** J Vermeij, Z Zeinoun, B Neyns, E Teugels, C Bourgain, J De Grève

**Affiliations:** Laboratory of Medical and Molecular Oncology and Oncology Center, Akademisch Ziekenhuis Vrije Universiteit, Brussel, Belgium; Department of Experimental Pathology, Akademisch Ziekenhuis Vrije Universiteit, Brussel, Belgium

**Keywords:** cancer gene therapy, recombinant adeno-associated virus, recombinant adenovirus, ovarian cancer

## Abstract

Recombinant adeno-associated virus (rAAV) vectors have emerged as vehicles for gene therapy. In addition, anti-neoplastic properties have been attributed to wild-type AAV. To take advantage of both features and to overcome technical problems associated with rAAV preparation, we developed a production method in which rAAV particles are amplified in an infectious cycle in the presence of wtAAV. This results in a 10^3^−10^4^-fold amplification of rAAV input particles. rAAV-GFP particles generated by this method were used to transduce ovarian cancer cell lines to evaluate their potential in ovarian cancer gene therapy, in comparison to a rAd-GFP vector. The transduction efficiency of NIH-OVCAR3, MDAH 2774 and SKOV3 cells with rAAV-GFP particles was low (< 1%) and did not improve by increasing the number of particles/cell. Repeated administration and continued exposure of NIH-OVCAR3 and MDAH 2774 improved transduction to over 3%. In contrast, these cell lines were more efficiently transduced by rAAV-GFP in the presence of adenovirus (~15%) and by rAd-GFP (> 50%). These results indicate that in contrast to rAd vectors, rAAV particles are not suitable for therapeutic gene transfer in ovarian cancer cells unless efficient help can be provided to mediate ss to ds DNA conversion.© 2001 Cancer Research Campaign  http://www.bjcancer.com


*British Journal of Cancer* (2001) **85**(10), 1592–1599
© 2001 Cancer Research Campaign
doi: 10.1054/ bjoc.2001.2082, available online at http://www.idealibrary.com on IDEAL

http://www.bjcancer.com

### Transduction of ovarian cancer cells: a recombinant adeno-associated viral vector compared to an adenoviral vector

**J Vermeij[1]*, Z Zeinoun[1], B Neyns[1]*, E Teugels[1], C Bourgain[2] and J De Grève[1]**

[1]Laboratory of Medical and Molecular Oncology and Oncology Center Akademisch Ziekenhuis Vrije Universiteit Brussel, Belgium; [2]Department of Experimental Pathology, Akademisch Ziekenhuis Vrije Universiteit Brussel, Belgium

**Summary** Recombinant adeno-associated virus (rAAV) vectors have emerged as vehicles for gene therapy. In addition, anti-neoplastic properties have been attributed to wild-type AAV. To take advantage of both features and to overcome technical problems associated with rAAV preparation, we developed a production method in which rAAV particles are amplified in an infectious cycle in the presence of wtAAV. This results in a $10^3$–$10^4$-fold amplification of rAAV input particles. rAAV-GFP particles generated by this method were used to transduce ovarian cancer cell lines to evaluate their potential in ovarian cancer gene therapy, in comparison to a rAd-GFP vector. The transduction efficiency of NIH-OVCAR3, MDAH 2774 and SKOV3 cells with rAAV-GFP particles was low (< 1%) and did not improve by increasing the number of particles/cell. Repeated administration and continued exposure of NIH-OVCAR3 and MDAH 2774 improved transduction to over 3%. In contrast, these cell lines were more efficiently transduced by rAAV-GFP in the presence of adenovirus (~15%) and by rAd-GFP (> 50%). These results indicate that in contrast to rAd vectors, rAAV particles are not suitable for therapeutic gene transfer in ovarian cancer cells unless efficient help can be provided to mediate ss to ds DNA conversion. © 2001 Cancer Research Campaign http://www.bjcancer.com

**Keywords**: cancer gene therapy; recombinant adeno-associated virus; recombinant adenovirus; ovarian cancer

Human epithelial ovarian cancer is the leading cause of death from gynaecological malignancies among women in the Western world. One out of 70 women develops the disease in the course of her life. No effective screening methods are currently available. Most women with ovarian cancer present with advanced disease at the time of diagnosis. Current standard therapeutic options include debulking surgery and adjuvant chemotherapy.

The poor long-term outcome of ovarian cancer calls for novel diagnostic as well as therapeutic strategies. One of these new therapeutic approaches is corrective gene therapy aiming to inhibit oncogene expression or to replace an inactivated tumour suppressor gene by a wild-type copy. Current clinical experimental gene therapy protocols (phase I and II) for ovarian cancer mostly use recombinant retro- and adenoviruses as vehicles for gene transfer (Barnes et al, 1997).

A promising viral vector is the recombinant adeno-associated virus (rAAV). This vector has characteristics that could be advantageous when compared to other commonly used vector systems. However, the potential of rAAV vectors in gene therapy for cancer has not been widely explored yet.

Wild-type (wt) AAV is not known to be pathogenic in humans and does not replicate without a helper virus. An interesting feature of the wild-type AAV is its tumour suppressive property (Schlehofer, 1994). In addition, wtAAV-infected cancer cells seem to be more sensitive to radio- and chemotherapy (Walz et al, 1992; Hillenberg et al, 1999). Recombinant AAV vectors are derived from the parental virus by deleting the *rep* and *cap* genes, which can be replaced by a transgene and its regulatory sequences necessary for expression. The only remaining sequences in *cis* in such constructs are the ITRs (Samulski et al, 1989). rAAV vectors have a broad host range including dividing as well as non-dividing cells, low immunogenicity and lead to stable expression of the transgene due to integration into the genome (Hallek and Wendtner, 1996).

The classic rAAV production procedure involves co-transfection of a plasmid containing a transgene flanked by the AAV ITRs and a complementor plasmid supplying the *rep*- and *cap* genes (without ITRs) into HEK-293 cells, followed by infection with adenovirus. Vectors are then concentrated and purified by 2 or 3 cycles of caesium chloride gradient ultra-centrifugation (Grimm et al, 1998). In order to reach high numbers of recombinant infectious particles, large-scale preparations need to be made, which makes this procedure labour intensive.

Despite several improvements in production as well as in concentration of rAAV vectors, this hurdle still remains (Gao et al, 1998; Grimm et al, 1998; Clark et al, 1999). These difficulties prompted us to explore an alternative production method consisting of 2 steps. The first step is a classical co-transfection which is followed by an amplification round of rAAV particles in the presence of wtAAV and adenovirus particles on HeLa cells. The presence of wtAAV, in the final rAAV stock was considered as potentially beneficial in light of its tumour suppressive properties. We explored the potential of this preparation of recombinant adeno-associated viral vectors and wtAAV to transduce human epithelial ovarian cancer cells. Transduction efficiency by this rAAV viral system was compared to a recombinant adenovirus vector.

*Received 5 February 2001*
*Revised 17 July 2001*
*Accepted 19 July 2001*

*Correspondence to:* J De Grève

*Both authors contributed to the work as Aspirant of the National Fonds voor Wetenschappelijk Onderzoek (FWO-Vlaanderen), Belgium.

### MATERIALS AND METHODS

### Cell lines

HeLa, HEK-293 and the epithelial ovarian cancer cell lines: SKOV3, NIH-OVCAR3 and MDAH 2774 were purchased from the ATCC (Manassas, VA, USA).

Cells were maintained in their recommended media (HeLa and HEK-293 cells: Dulbelcco's modified Eagle's medium (DMEM), supplemented with 10% fetal calf's serum (FCS), 50 IU ml$^{-1}$ penicillin (P) and 50 µg ml$^{-1}$ streptomycin (S), SKOV3: McCoy's 10% FCS P/S, NIH-OVCAR3: RPMI 20% FCS P/S, MDAH 2774: Leibovitz 10% FCS P/S.

293pTP cells were a gift from Dr J Schaack, University of Colorado, Denver, USA. Cells were maintained in DMEM 10% FCS P/S and hygromycin 25 µg ml$^{-1}$ (Schaack et al, 1996; Maxwell et al, 1998).

Media and supplements were supplied by GIBCO, Life Technologies, Gaithersburg, MD, USA.

Cells were grown in a humidified chamber with 5% $CO_2$ at 37˚C.

### Production of viruses

1. rAAV-GFP particles were generated by co-transfection of HEK-293 cells (5 × 10$^6$ cells/10-cm plate) with the pTR-UF2 plasmid which contains the humanized form of green fluorescent protein (GFP) (Zolothukin et al, 1996) and the pIM45 plasmid which contains the AAV *rep* and *cap* sequences (map positions: 190 – 4489). Lipofectamine was used according to the suppliers instructions (GIBCO). 6 hours after transfection, cells were infected with wild-type adenovirus (MOI 1) for 2 hours. (pTR-UF2 and pIM45 were gifts from N Muzyczka and SL Zolothukin, University of Florida and L Tenenbaum, Université Libre de Bruxelles.)

   After 72 h a lysate was collected and treated by 3 cycles of freeze/thawing, followed by heat inactivation of adenovirus (30 min, at 56˚C). This crude lysate was centrifuged (Beckmann) at 8000 rpm for 15 min at 4˚C. Afterwards the supernatant was passed through a 0.20 µm filter (Sartorius, Goettingen, Germany), aliquoted in 2 ml fractions and stored at –80˚C.
2. rAAV-GFP particles were then amplified by co-infecting HeLa cells (5 × 10$^6$ cells/10 cm plate) with equal amounts of rAAV-GFP and wtAAV infectious particles (10$^3$/10$^3$) followed by adenovirus infection at a MOI of 1. After 3 days the lysate was collected and treated as described above. These rAAV-GFP particles (amplified stock) were subsequently concentrated, purified by column affinity chromatography using Cellufine sulfate media (Amicon Division, WR Grace & Co, Danvers, MA, USA) according to Tamayose et al (1996). A 35 × 115 mm column was used. Sample (amplified rAAV-GFP crude lysate, titre around 10$^6$ IP ml$^{-1}$) was loaded on the column, followed by washing with PBS, until absorbance readings at 280 nm were below 0.01 (GeneQuant, Pharmacia, Cambridge, England). The particles were eluted by 1.0 M NaCl solution. Individual fractions (3.5 ml) were tested for the presence of infectious particles (by in situ replication centre assay). The final numbers of particles in separate preparations were in the range of 10$^7$–10$^8$. The particles were stored at –80˚C after dialysis (Slide-A-Lyzer, Pierce, Rockford, II, USA) against PBS for use in transduction experiments. No rAAV-GFP particles could be detected in the run through fractions during sample loading. Recovery was around 50%. The ratio wtAAV/rAAV was unaffected by chromatography.
3. Wild-type AAV 2 was generated by transfection of HEK-293 cells with pSM620 (containing the entire wild type AAV genome, a kind gift from P Hermonat, University of Arkansas, Little Rock, USA) and lipofectamine, followed by adenovirus infection at a MOI of 5.

   The crude lysate was harvested after 3 days and treated as described above.
4. Adenovirus serotype 2 (wt Ad) was propagated in HEK-293 cells, the lysate was collected after full cytopathic effect, freeze/thawed 3 times and purified by caesium chloride gradient ultracentrifugation. An opalescent band at 1.33 g ml$^{-1}$ was recovered and dialysed against PBS. The concentrate was diluted 1:2 in TRIS-HCl pH 8.0-Glycerol 50%.
5. Ad5del308ΔpTP (again a gift from Dr J Schaack) was amplified in complementing 293pTP cells at a MOI > 10. After 3 days the lysate was collected, freeze/thawed 3 times and centrifuged at 8000 rpm at 4˚C for 15 minutes. A titre of 10$^6$IP ml$^{-1}$ lysate was obtained.
6. Recombinant adenoviral particles, rAd-GFP were generated, purified and titrated according to the protocol described by T-C He et al (1998) and available at www.coloncancer.org. The necessary plasmids were obtained with permission of B Vogelstein (John Hopkins, Baltimore). The final preparation was stored at –20˚C. Titres were determined by transduction on HEK-293 cells after 18 hours (1 – 2 × 10$^8$ efu ml$^{-1}$). Based on these titres MOIs for rAd-GFP were determined. No wild-type adenovirus could be detected in the stock neither by PCR (for Ela region) nor by transfer experiments.

### Titration of rAAV (crude lysate) stocks

Three methods were used:

1. Dot blot hybridization assay. A dot blot assay was performed as described in Basic Methods in Molecular Biology (Davis et al, 1986, p 147–149). All DNA samples were applied on nitrocellulose membranes (Hybond; Amersham, Buckinghamshire, England) by a Biorad blotting apparatus (Hercules, CA, USA). The membranes were hybridized with P$^{32}$ labelled PCR-based specific probes. Titres were estimated through comparison of the sample signals with standard plasmid DNA dilutions (Phospho-imager, Biorad).
2. Transduction assay. 5 × 10$^5$ HeLa cells well$^{-1}$ were plated in a 6-well plate. The next day the cells were infected with rAAV-GFP particle stock (co-transfection or amplified) and wtAAV plus Ad virus were added, in DMEM 2% FCS. After 2 h the supernatant was removed and DMEM 10% FCS was added. 48 h after infection the number of green cells was counted using an AXIOVERT 25 fluorescence microscope (Zeiss, Oberkochen, Germany).
3. In situ replication centre assay (RCA). Briefly, 5 × 10$^5$ HeLa cells well$^{-1}$ were seeded. The next day cells were infected with serial dilutions of rAAV-GFP particle stock and wtAd in DMEM 2% for 2 hours. WtAAV was added only when rAAV-GFP particles were titrated. 24 h after infection the cells were treated as described by L Tenenbaum et al (1999). Briefly, after removal of the medium, cells were washed twice. Nitrocellulose membranes were then firmly pushed on the

© 2001 Cancer Research Campaign 

cells and wetted with buffer (Tris 1M pH 8), which lyses the cells, followed by denaturation with NaOH 0.5 M NaCl (3 × 1 min), neutralization with NaCl 1.5 M Tris 0.5 $M^{-1}$ pH 8 (3 × 1 min) and washing with SSC 2 × (3 × 1 min). After baking the membranes for 2 h at 80˚C, they were hybridized overnight with $P^{32}$-labelled probes. The probes were PCR-based and recognize a 362 bp sequence of GFP (bases 136–498) or a 516 bp rep sequence (bp 753–1269). Replication centres were counted after autoradiography and the viral infectious particle (IP) titres were calculated taking into account the dilution factors. Whenever the number of individual centres could not be estimated due to high density, phospho-imaging was used for quantification.

### Infection of ovarian cancer cell lines

*Infection with rAAV-GFP*

NIH-OVCAR3, SKOV3, MDAH 2774 and one control cell line (HeLa) (1 × $10^4$ cells $well^{-1}$, in triplicate) were infected with purified rAAV-GFP particles at MOI 1, 10 and 100 (IP $cell^{-1}$). PBS without particles (mock-infected), wtAAV infection at MOI 10 IP $cell^{-1}$ and untreated cells served as controls. After 18–24 h the supernatant was removed and replaced by fresh medium. In a second set of conditions rAAV-GFP particles were administered repeatedly (MOI 1 IP $cell^{-1}$, once daily for 5 days) without replacement of the supernatant and compared to single administration of rAAV-GFP at MOI 1 (without replacement of supernatant). Controls included mock (PBS) and wt AAV infections in similar conditions. Transduction was evaluated daily using an Axiovert 25 fluorescence microscope (Zeiss) for 10 days.

Another set of experiments consisted of co-infecting these cell lines with rAAV-GFP and adenovirus (wtAd or Ad5del308ΔpTP) for 2 hours in serum-free medium which was then replaced by serum-containing medium. GFP expression was evaluated daily for 4–6 days.

Cell counts were performed using an ocular with a counting frame (area 100 squares). Total cell numbers were calculated based on at least 3 different counts of minimal 10 squares $well^{-1}$.

*Infection with rAd-GFP*

The same set of epithelial ovarian cancer cell lines was used to evaluate transduction efficiency by rAd-GFP vectors. Again infection at increasing MOIs (1–10 –100 expression forming units cell: (efu)/cell) was tested. GFP expression was analysed as described above. Controls included uninfected cells and particle storage buffer alone in a volume equal to the infected cells.

### Immunocytochemistry

After fixation in formaldehyde 4% cells were subsequently exposed to: (1) $H_2O_2$ 0.1% in methanol 10 min; (2) normal goat serum + BSA for 20 min; (3) polyclonal rabbit anti-GFP (Santa-Cruz, Santa-Cruz, Ca, USA) dilution of 1/400 at 4˚C overnight; (4) goat anti-rabbit biotin 1/300 (Amersham-Pharmacia, Roosendaal, Nl), 30 min at room temperature; (5) streptABComplex/HRP (DAKO, Glostrup, Denmark), 30 min at room temperature; and (6) diaminobenzidine (DAB) 5 min at room temperature (Sigma-Aldrich, Bornem, Belgium). A more intense staining was obtained by $CuSO_4$ followed by counter-staining with haematoxylin. Slides were dehydrated and mounted. Sections were evaluated and photographed under an Olympus BX 41 microscope using a DP-11 digital camera (Olympus, Hamburg, Germany).

### RESULTS

### Generation of recombinant AAV-GFP vectors by wt AAV assisted amplification on HeLa cells

A 2-step strategy for generation of high-titre rAAV preparations was developed. Plasmid co-transfection of HEK-293 cells for generation of rAAV particles on a small scale was followed by amplification of these rAAV particles on HeLa cells with wtAAV as a complementor virus and adenovirus as a helper virus. On average non-purified rAAV stocks made by co-transfection contained $10^4$ infectious recombinant adeno-associated virus particles $ml^{-1}$ and were free of detectable wtAAV. These titres are too low for use in in vitro and in vivo experiments in which a MOI > 1 is to be used. Amplification of rAAV-GFP particles in the presence of wtAAV and adenovirus was investigated on 2 cell lines (HeLa and HEK-293), in an attempt to increase the yield of recombinant AAV-GFP particles. Yields of infectious rAAV-GFP particles on HeLa cells were higher when compared to HEK-293 cells. The highest amplification factor on HeLa cells was obtained when rAAV- and wtAAV-infectious particles were added in equal amounts.

This approach led consistently to an amplification of rAAV particles on HeLa cells by $10^3$–$10^4$ fold, when compared to the initial amount of infectious rAAV particles. Amplification on HEK-293 cells was 5–10-fold lower.

The rAAV (amplified) stocks generated with this method were further characterized. The titres of rAAV particles as well as wt AAV particles were estimated by dot blot (genomic particles), transduction (rAAV)- and in situ replication centre assay (Table 1). Titres derived from in situ replication centre assay (infectious

**Table 1** Characterisation of crude lysate of amplified rAAV-GFP stocks

| | Particles $ml^{-1}$ | | |
|---|---|---|---|
| **Titration method** | **rAAV-GFP** | **Ad 2** | **wtAAV** |
| Dot blot assay | 3.2 $10^9$ +/– 2.2 $10^9$ | N.D | 1.4 $10^{10}$ +/– 1.8 $10^9$ |
| Transduction assay | 3.6 $10^7$ +/– 2.8 $10^7$ | – | – |
| In situ replication assay | 9.5 $10^5$ +/– 6.1 $10^5$ | 0 | 2.7 $10^7$ +/– 1.4 $10^7$ |

rAAV-GFP preparations obtained by amplification in HeLa cells, were characterised for their biological activity (transducing units and infectious particle number) and their physical contents (genomic particle number by dot blot assay) before concentration by column chromatography. The amount of rAAV-GFP, wtAAV and Ad 2 particles was determined. Data presented are the average (+/– SD) of results obtained with at least three separately prepared rAAV-GFP stocks. N.D = not determined.

*© 2001 Cancer Research Campaign*

particles ml$^{-1}$) were used to calculate the volume needed to infect cells at different MOIs in subsequent experiments. Column chromatography (Tamayose et al, 1996) was performed to concentrate and purify the stocks.

The ratio between infectious and genomic rAAV particles was estimated to be 1 to 100–1000 in these preparations. The ratio between rAAV-GFP and wtAAV particles was 1/10–100.

After adenovirus heat inactivation and column purification, no replicative adenoviral particles could be detected in the rAAV-GFP stocks by in situ replication centre assay hybridizing with an adenoviral probe.

### Transduction efficiency of human epithelial ovarian cancer cell lines with rAAV vectors in vitro

3 ovarian cancer cell lines: NIH-OVCAR3, SKOV3, MDAH 2774 and HeLa cells were infected with purified rAAV-GFP particles from an amplified stock to study the transduction efficacy of rAAV vectors on epithelial ovarian cancer cells. A number of experimental conditions were studied. Single administration at increasing MOIs of 1, 10 and 100 was examined on these 4 cell lines. Repeated administration was evaluated on NIH-OVCAR3 and MDAH 2774 cells, at a MOI of 1 for a 5-day period. Also, continued exposure of the cells to rAAV-GFP particles was studied on these 2 cell lines.

Under all of these circumstances transduction efficiencies were extremely low (< 5%) (Table 2). Neither did they improve with increasing MOIs (10–100). There was only a minor improvement (from < 1% to 3%) in transduction efficiency when NIH-OVCAR3 cells were continuously exposed to rAAV-GFP particles at a MOI of 1. The effect of which was equal to repeated administration at the same MOI.

GFP transduction of MDAH 2774 cells, transferred to a larger dish on day 4 after infection suddenly increased on day 6 yielding maximum transduction percentages of 2.8% (MOI 1), 1.3% (MOI 10) and 2.3% (MOI 100) on day 7. However simultaneously, rAAV-GFP-infected MDAH 2774 cells also started to round up and detach, whereas all controls continued to grow. The supernatant of all cell lines taken at day 8 was analysed for the presence of adenovirus. In situ replication centre assay after hybridizing with an adenoviral probe revealed unexpectedly the presence of adenovirus in the supernatant of the MDAH 2774 cell line, while no signal could be detected in the supernatant of the other cell lines (results not shown). This suggests presence of a small, undetected amount of wild-type adenovirus in the rAAV-GFP stock used and which under these experimental conditions is able to proliferate in MDAH 2774 cells to a detectable level.

Consistent with previous reports, HeLa cells also proved difficult to transduce with purified rAAV-GFP particles, resulting in very low to absent GFP expression (Qing et al, 1997, 1998; Tenenbaum et al, 1999).

MDAH 2774, SKOV3 and HeLa cells were not much influenced in their growth by the infection with rAAV-GFP or wtAAV. All conditions (including controls) reached confluence between days 3–4.

In contrast, in the NIH-OVCAR3 cell line a cytopathic effect was observed after infection with the rAAV-GFP particles as well as with wtAAV (MOI 10) infection alone, leading to 100% cell death by day 7 in rAAV-GFP-infected cells (condensed nuclei and detached cells). Only 30% of the wtAAV-infected cells survived, whereas mock-infected and untreated cells continued to grow and reached confluence by the end of the observation period. This effect on the cell number seemed to be independent of the MOIs (1–5–10–50) used for both rAAV/wtAAV infection as well as wtAAV infection (data not shown).

### Effect of adenovirus on the transduction efficiency of ovarian cancer cells

The low transduction efficiency of ovarian cancer cells could be due to either problems of recombinant viral particle entry or inefficient intracellular processing and expression of the vector. To elucidate this, wild-type adenovirus (MOI 0.1) was added to the 3 ovarian cancer cell lines together with rAAV-GFP particles at a low MOI (0.1).

Under these conditions GFP expression was clearly observed within 24 hours in the cell lines by fluorescence microscopy and by immunocytochemistry, used as an additional means to demonstrate transduction (Figure 1, right panel). It has been reported (Brewis et al, 2000) in other settings that this method is more sensitive than direct fluorescent microscopy for GFP detection.

Only very few cells were stained in absence of adenovirus after 72 h (Figure 1, left panel), when exposed to rAAV-GFP at an MOI of 1. The finding of GFP-positive cells in the presence of adenovirus

**Table 2** Maximal transduction efficiency (%) of rAAV-GFP particles on ovarian cancer cell lines

| | | rAAV-GFP IP/cell | | | | |
|---|---|---|---|---|---|---|
| Cell lines | | MOI 1 | MOI 10 | MOI 100 | MOI 1/daily × 5 | MOI 1 continued exposure |
| SKOV3 | – | <1 | <1 | <1 | N.D | N.D |
| MDAH 2774 | – | <1 | <1 | <1 | <1 | <1 |
| | + | 2.1 +/– 1.5 | N.D | N.D | N.D | N.D |
| HeLa | – | <1 | <1 | <1 | N.D | N.D |
| | + | 7.4 +/– 1.0 | N.D | N.D | N.D | N.D |
| NIH-OVCAR3 | – | <1 | <1 | <1 | 3.3 +/– 1.3 | 3.3 +/– 0.9 |
| | + | 13.0 +/– 1.8 | 13.6 +/– 4.6 | 17.2 +/– 3.3 | N.D | N.D |

Maximum transduction efficiency (%) in time, after infection of, SKOV3, MDAH 2774, HeLa and NIH-OVCAR3 cells with rAAV-GFP particles at increasing MOI's (1, 10, 100 transient exposure) or with repeated administration at a MOI of 1 for 5 days or continuous exposure at MOI 1. The effect on transduction with (+) and without (–) co-infection at MOI 1, with wild type or a mutant (replication defective) adenovirus (Ad5del308ΔpTP) is shown. Transduction efficiency was calculated as the percentage of GFP positive cells over the total cell number. Observation period: ten days. Green fluorescent cells were present on day three (without adenovirus) and within 24 hours with adenovirus. Data presented are as the mean +/– SD of three experiments. MOI's are defined in infectious particles/cell, based on titers determined by in situ replication center assay. N.D: not done.

*© 2001 Cancer Research Campaign* 

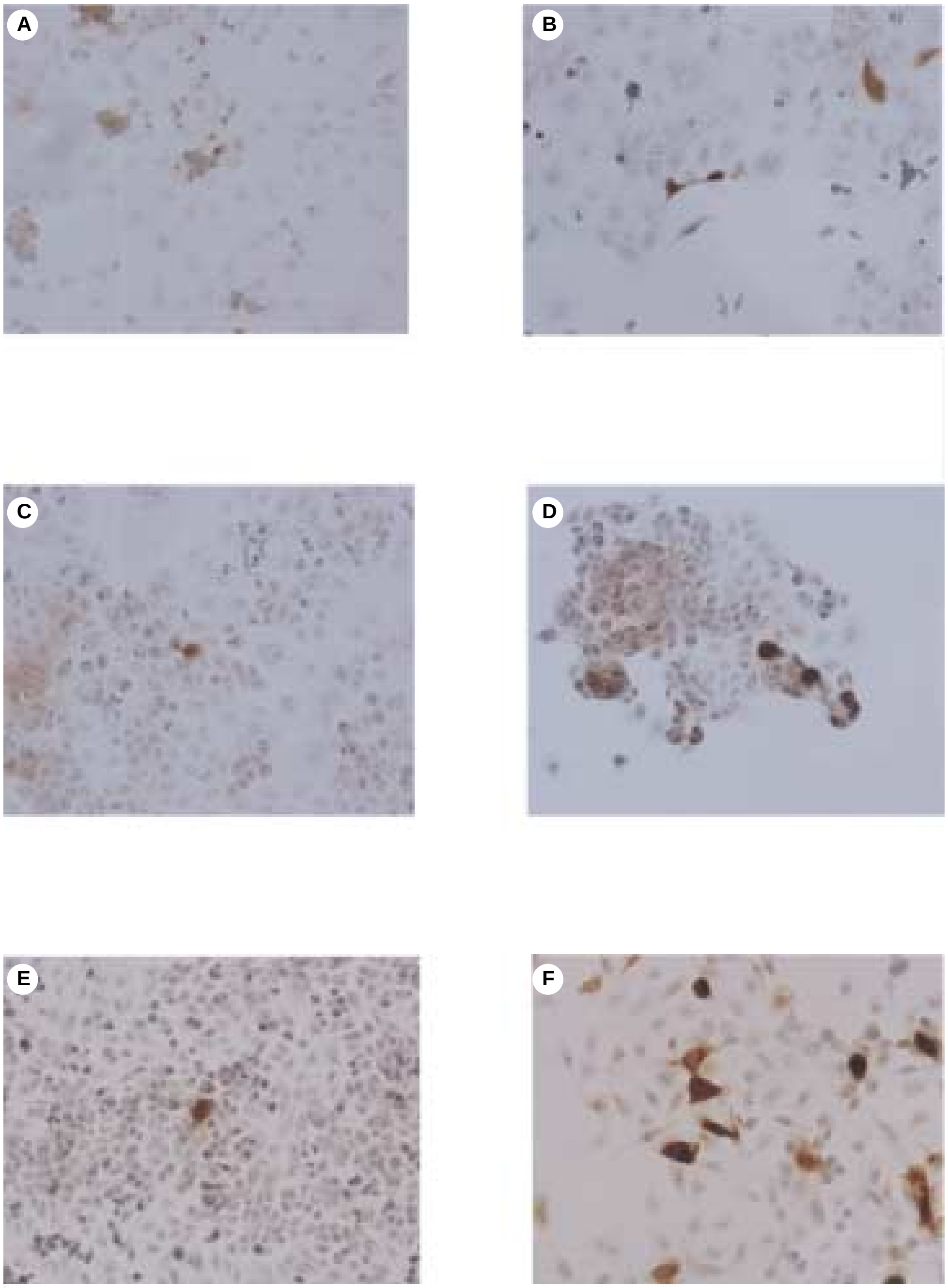

**Figure 1** Immunocytochemistry for GFP on MDAH 2774 (**A**,**B**), NIH-OVCAR3 (**C**,**D**) and HeLa (**E**,**F**) cells. Cells were infected with rAAV-GFP at a MOI of 1 (I.P/cell) in the absence of wild-type adenovirus (**A**,**C**,**E**) and at a MOI of 0.1 (I.P/cell) in the presence of wild type adenovirus (**B**,**D**,**F**). Cells were fixed after 24 h (**B**,**D**,**F**) and 72 h (**A**,**C**,**E**). Magnification 200 ×, counterstaining with haematoxylin coincided with the finding of replication centres after in situ hybridization of the cell lines with a GFP probe (Figure 2). In the absence of adenovirus, these centres were not found and green fluorescent cells were absent. This is an indication that replicative (double-stranded) forms of rAAV-GFP DNA are needed for expression and adenovirus helps to generate these forms in the cell lines examined. A similar observation has already been made by others for different cell types (Ferrari et al, 1996; Fisher et al, 1996; Peng et al, 2000). Help from wild-type adenovirus can only be observed for a limited time, due to its replication which causes a cytolytic effect within 72 hours after infection. Therefore a mutant, replication-defective adenovirus (Ad5del308ΔpTP, E1 and E4ORF6 expressed) was also evaluated. Since this adenovirus mutant does not replicate, cell death was far less dramatic when compared to wild-type adenovirus co-infection and observations could be extended over longer periods (5–6 days).

To further assess the transduction efficiencies in the presence of adenovirus MDAH 2774, HeLa and NIH-OVCAR3 cells were co-infected with rAAV-GFP (MOI 1) and Ad5del308ΔpTP or wt adenovirus (MOI 1). A slight increase in expression of GFP in MDAH 2774 (2.1%) and HeLa (7.4%) could be observed but a more significant increase was only found in NIH-OVCAR3 cells

 *© 2001 Cancer Research Campaign*

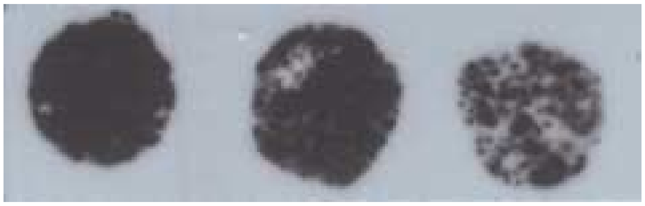

1 2 3

**Figure 2** NIH-OVCAR 3, SKOV 3 and MDAH 2774 ovarian cancer cells coinfected with rAAV-GFP (MOI < 1 I.P) and (wild-type) adenovirus. Viral particles were removed after 2 hours and cells were given fresh medium. The in situ replication centre assay was started 28 hours after infection. Membranes were hybridized with a $P^{32}$-labelled GFP probe. Autoradiography was developed after 4 hours. Without adenovirus present no signal could be detected, even after long expositions (48 hours, data not shown). The presence of replication centres coincided with detection of green fluorescent cells.
(1) NIH-OVCAR 3 (2) MDAH 2774 (3) SKOV 3

(13%) (Table 2). Therefore, in addition, the effect of increasing the MOIs of rAAV-GFP (MOI 10–100) was studied on these cells (Table 2, NIH-OVCAR3 + Ad5del308ΔpTP). Transduction efficiencies in a range similar to rAAV-GFP MOI 1 were found, indicating a limitation in transduction again independent of rAAV-GFP concentration.

### Transduction efficiency of epithelial ovarian cancer cell lines with rAd-GFP in vitro

The transduction efficiencies observed with the recombinant adeno-associated virus system in ovarian cancer cells, even in the presence of appropriate help, such as wt or mutant adenovirus or genistein (data not shown) are too low for gene transfer when therapeutic genes are to replace GFP. In corrective gene therapy for example a near 100% transduction efficiency would be needed. We therefore compared the rAAV-GFP system to the recombinant adenoviral vector system which has already been used in clinical protocols and evaluated the transduction efficiency of this vector on the same epithelial ovarian cancer cell lines (Table 3).

In general, transduction efficiencies with rAd-GFP were much higher in all cell lines examined, reaching more then 50% within 48 hours when a MOI of 10 was used. There was a clear dose–response effect.

**Table 3** Maximal transduction efficiency (%) of rAd-GFP particles on ovarian cancer cell lines

| | rAd-GFP (efu cell$^{-1}$) | | |
|---|---|---|---|
| **Cell lines** | **MOI 1** | **MOI 10** | **MOI 100** |
| NIH-OVCAR3 | 34.0 +/– 8.2 | 50.8 +/– 6.3 | toxic |
| SKOV3 | 2.9 +/– 1.8 | 60.0 +/– 13 | toxic |
| MDAH 2774 | 8.3 +/– 1.7 | 71.1 +/– 1.8 | toxic |
| HeLa | 21.6 +/– 7.5 | 51.1 +/– 9.4 | toxic |

Maximum transduction efficiency (%) in time after infection of NIH-OVCAR3, SKOV3, MDAH 2774 and HeLa cells with rAd-GFP particles, at increasing MOI's (1, 10, 100 continuous exposure). Transduction efficiency was calculated as the percentage of GFP positive cells over the total cell number (three representative areas/well). Experiments were done in triplicate. Observation period: five days. Green fluorescent cells were present 18 hours after infection. Data presented are as the mean +/– SD.
MOI's are defined in expression forming units/cell, based on titers determined with the transduction assay on HEK-293 cells (see Methods).

Cytotoxic effects were observed in all cell lines. MOIs of 100 or more caused a lethal effect in the 4 cell lines within 18 hours. This effect is not vector- or GFP-related, since GFP expression was absent at this time and a similar effect could be observed when equal volumes of just particle storage buffer (glycerol-containing) were put on the cells.

At a MOI of 10 a growth inhibitory effect was noticed after 48 hours of infection in all cell lines. By then the cells were already strongly expressing GFP. This was not observed in control conditions in which the cells only received storage buffer. To exclude the presence of wild-type adenovirus, PCRs for the E1a region were performed on the supernatants of the cells as well as transfer experiments. From these tests there was no evidence that wtAd virus was responsible for these effects. Most likely the inhibition is due to the rAd vector itself or to the GFP (Lui et al, 1999).

### DISCUSSION

Over the past few years recombinant adeno-associated viruses have gained interest as vectors for gene transfer in human gene therapy (Hallek and Wedntner, 1996). Production methods for recombinant adeno-associated virus have improved (Grimm et al, 1998) but there is still no uniform production method and most procedures are laborious because they need to be performed on a large scale to obtain enough viral particles for in vivo experiments.

In this paper we report our exploration of an alternative approach for the production of rAAV particles, for use in cancer gene therapy. The strategy involves a 2-step procedure in which rAAV particles generated by co-transfection (on a small scale) are subsequently amplified on HeLa cells in the presence of wild-type AAV virus and adenovirus, followed by concentration using column chromatography. Our results demonstrate that this procedure is a less laborious alternative with a high amplification factor ($10^3$–$10^4$) for production of rAAV stocks when compared to large-scale co-transfection methods.

The final stocks obtained by our method contain an excess of wild-type AAV particles. For corrective cancer gene therapy this would not necessarily be a disadvantage since tumour-suppressive properties have been attributed to wild-type adeno-associated virus (Schlehofer, 1994). In addition there have been reports demonstrating an inhibiting effect of the AAV rep gene on cellular transformation (Khleif et al, 1991; Kube et al, 1997; Batchu et al, 1999). Wild-type AAV infection has also been found to increase the sensitivity of malignant cells to chemotherapy and radiotherapy; this could be an advantage when treatment modalities are combined (Walz et al, 1992; Hillgenberg et al, 1999). There is however a necessity to use wtAAV as a control in experimental settings in order to be able to identify the specific effect of the therapeutic insert within the rAAV vector.

There are only limited data on the efficiency of rAAV vectors for gene transfer into human cancer cells (Hoerer et al, 1997; Maass et al, 1998; Veldwijk et al, 1999). We studied the transduction efficiency of rAAV-GFP vectors on 3 ovarian cancer cell lines in vitro.

The basic transduction efficiency with rAAV-GFP/wtAAV in all ovarian cancer cell lines in our study was very low. The transduction efficiency on these cells could however be improved in various ways: to a minor extent with continuous exposure of the cells to rAAV-GFP or by repeated administration as studied on NIH-OVCAR3 cell line. With the help from adenovirus (wild-type or mutant) a larger improvement in transduction efficiencies was observed, as reported by others (Ferrarri et al, 1996; Fisher et al,

*© 2001 Cancer Research Campaign*

1996; Qing et al, 1997, 1998; Mah et al, 1998). On NIH-OVCAR3 cells this increase also proved to be independent of the rAAV-GFP concentration. Data published on transduction of hepato-cellular carcinoma with rAAV are comparable to our findings (Peng et al, 2000). These authors also observed very low transduction efficiencies when infecting several HCC cell lines with rAAV containing a reporter gene at increasing MOIs. The transduction efficiency could be increased with the help of adenovirus as well as γ-radiation and chemotherapy.

The absence of an important dose–dependent effect and the efficient transduction at low MOI in the presence of wild-type or mutant adenovirus indicate that post-entry events rather than viral uptake limit transduction efficiency of rAAV-GFP in these ovarian cell lines.

A possible explanation for the low transduction efficiencies observed could be suppression of translation of rAAV vectors due to the presence of the rep 78 protein from the wtAAV in the preparation (Takeuchi et al, 2000). This however seems less likely. We performed small-scale in vitro experiments with rAAV particles generated by co-transfection (these stocks are wtAAV-free) and the same low transduction efficiency was obtained (data not shown).

Moreover the experiments by Peng et al on hepatocellular carcinoma (HCC) were also performed with rAAV particles free from wtAAV.

The higher transduction efficiencies observed in ovarian carcinoma cells tested by Maass et al could be due to various experimental variables: cell types tested; freshly isolated tumour cells versus cell lines; use of luciferase vs GFP as a reporter gene, which have different detection methods (cell extracts vs life cells). In addition in all experiments reported by Maass, the cells were exposed to X-ray irradiation causing an increase in transduction comparable to that obtained with an adenovirus super-infection at a MOI of 10, while we only used a MOI of 1.

The major hurdle for transduction by rAAV vectors, after entry of the single-stranded DNA rAAV particles is the conversion to a transcription-competent double-stranded DNA (Samulski et al, 1989; Fisher et al, 1996; Peng et al, 2000). Several cellular factors play a role in this conversion. Recent publications could demonstrate a correlation between the transduction efficiency by rAAV vectors and the phosphorylation status of a cellular tyrosine phospho-protein (ssD-BP) (Ferrari et al, 1996; Qing et al, 1997, 1998; Mah et al, 1998). This protein binds to the 3′ end of the AAV genome. Transduction by AAV is related to the ratio between the dephosphorylated and phosphorylated form of this protein. Different cell types and tissues seem to have different ratios. No specific data on the ratio between dephosphorylated and phophorylated ssD-BP in ovarian cancer cells or human tumours are currently available. Our data are compatible with a role for the phosphorylated status of ssD-BP in the ovarian cancer cell lines used in this study. Wild-type adenovirus and mutant adenovirus (Ad5del308ΔpTP), which are known to increase the dephosphorylated/phosphorylated ratio of ssD-BP, mainly through the E4ORF6 protein, led to a higher GFP expression in these ovarian cancer cells. At least a 5-fold increase in NIH-OVCAR3 transduction compared to rAAV-GFP infection alone could be observed.

The cytotoxicity observed in NIH-OVCAR3 and MDAH 2774 cells but not in SKOV3 and HeLa after exposure to rAAV-GFP/wtAAV preparations should be discussed.

Whereas SKOV3 and HeLa cells were unaffected in their growth, a lethal toxicity was observed in NIH-OVCAR3. In NIH-OVCAR3 cells growth arrest and subsequent cell death eventually took place in all conditions of viral infection (after the time points on which GFP transduction was measured), suggesting an effect of wtAAV since replicating adenovirus could not be detected in the supernatants of infected cells.

This cytopathic effect on NIH-OVCAR3 cells was independent of either the MOIs of rAAV/wtAAV or wtAAV preparations used and resembles the influence of wtAAV-2 infection on carcinoma cells described by Bantel-Schaal (1990) regarding cell number and morphologic alteration of these cells.

It appears that NIH-OVCAR3 cells are so sensitive to these toxic effects that even at the lowest MOI a maximal toxic effect can be observed which explains the absence of a dose–response effect.

Kube et al (1997) reported a growth arrest on primary human fibroblasts related to the presence of rep proteins associated and encapsidated in mature rAAV particles. This effect was MOI-dependent, however differences in susceptibility to AAV among tumour cell types have been described and are most likely due to different molecular pathways by which cells have become immortal (Kube et al, 1997).

The AAV-associated cytopathic effect was only observed on the NIH-OVCAR3 cell line and not on MDAH-2774 and SKOV3, although transduction efficiency of these cell lines was also low. Therefore the poor transduction efficiency observed in all 3 ovarian cancer cell lines could not be explained by the AAV-associated toxicity.

The late-onset cell death observed in the MDAH 2774 cells is probably due to a lytic effect of contaminating adenovirus. The presence of replicating wild-type adenovirus in the supernatant of infected cells can explain the final sudden increase in transduction observed in MDAH 2774 cells just before cell death occurred. The presence of adenovirus is probably due to a small number of adenoviral particles (initially below the detection limit of the in situ replication centre assay) in the original rAAV-GFP stock and to unknown factors specific for the MDAH 2774 cell line which favour adenovirus replication.

Because of the low transduction efficiencies obtained with rAAV, we wanted to compare this system to rAd transduction on the same ovarian cancer cells. The rAd vectors are easier to produce in large quantities and have already been used in clinical protocols, including ovarian cancer. Infection of ovarian cancer cells with rAd-GFP led to much higher transduction efficiencies that were dose-dependent and independent of additional help. Some cytopathic effects observed at lower MOIs could be explained by toxic effects of the adenoviral proteins or even of the recombinant GFP protein (Liu et al, 1999).

In conclusion, based on the features of rAAV vectors we wanted to explore their potential as vectors in corrective gene therapy for epithelial ovarian cancer. However the production of large amounts of rAAV particles has remained cumbersome. We tried to overcome this hurdle by using the method proposed in this article. This procedure proved to be an efficient and labour friendly way to generate rAAV particles. We then used them to evaluate transduction efficiencies in epithelial ovarian cancer cells. We found that ovarian cancer cells can be infected with rAAV vectors but significant expression of the transgene needs help from either adenovirus or other enhancers of transduction which constitutes an important obstacle for their application in vivo. In comparison rAd vectors, easier to generate, led to high transduction percentages in ovarian cancer cells in this study. For corrective therapeutic gene

 *© 2001 Cancer Research Campaign*

therapy strategies in human cancer a high transduction efficiency is critical. Therefore, adenoviral vectors currently remain the vectors of choice for gene transfer into human epithelial ovarian cancer cells.

### ACKNOWLEDGEMENTS

The authors would like to thank L Tenenbaum for supplying the pIM45 plasmid as well as S Zolothukin and N Muzyczka for providing us with pTR-UF2 and J Schaack for the mutant adenovirus Ad5del308ΔpTP and its complementing cell line. Thanks to G Van Hassel and N Buelens for excellent technical assistance. The work is supported by the Fonds voor Wetenschappelijk Onderzoek Vlaanderen (Grant number: # 3.0024.94, # 45761 and # G.0154.01) and de Sport Vereniging tegen Kanker.

### REFERENCES

Bantel-Schaal U (1990) Adeno-associated parvoviruses inhibit growth of cells derived from malignant human tumors. *Int J Cancer* **45**: 190–194

Barnes MN, Deshane JS, Rosenfeld M, Siegal GP, Curiel DT and Alvarez RD (1997) Gene therapy and ovarian cancer: a review. *Obstet Gynecol* **89**: 145–155

Batchu RB, Shammas, MA, Wang JY and Munshi NC (1999) Interaction of adeno-associated virus rep78 with p53: Implications in growth inhibition. *Cancer Res* **59**: 3592–3595

Brewis N, Phelan A, Webb J, Drew J, Elliott G and O’Hare P (2000) Evaluation of VP22 spread in tissue culture. *J Virol* **74**: 1051–1056

Clark KR, Liu X, McGrath JP and Johnson PR (1999) Highly purified adeno-associated virus vectors are biologically active and free of detectable helper and wild-type viruses. *Hum Gene Ther* **10**: 1031–1039

Davis LG, Dibner MD and Battey JF (1986) “Dot Blot” hybridization of labeled probe to DNA or RNA samples. In: *Basic methods of molecular biology*, Davis LG (ed) pp147–149. Elsevier: New York

Ferrari FK, Samulski T, Shenk T and Samulski RJ (1996) Second strand synthesis is a rate limiting step for efficient transduction by recombinant adeno-associated virus vectors. *J Virol* **70**: 3227–3234

Fisher KJ, Gao G-P, Weitzman MD, DeMatteo R, Burda JF and Wilson JM (1996) Transduction with recombinant adeno-associated virus for gene therapy is limited by leading strand synthesis. *J Virol* **70**: 520–532

Gao G-P, Qu G, Faust L, Engddahl RK, Xiao W, Hughes JV, Zoltick PW and Wilson JM (1998) High titer adeno-associated viral vectors from a rep/cap cell line and hybrid shuttle virus. *Hum Gene Ther* **9**: 2353–2361

Grimm D, Kern A, Rittner K and Kleinschmidt JA (1998) Novel tools for production and purification of recombinant adeno-associated virus vectors. *Hum Gene Ther* **9**: 2745–2760

Hallek M and Wendtner C-M (1996) Recombinant adeno-associated virus (rAAV) vectors for somatic gene therapy: recent advances and potential clinical applications. *Cytokines and Mol Ther* **2**: 69–79

He T-C, Zhou S, da Costa LT, Yu J, Kinzler KW and Vogelstein B (1998) A simplified system for generating recombinant adenoviruses, *Proc Natl Acad Sci USA* **95**: 2509–2514

Hillenberg M, Schlehofer JR, von Knebel Doeberitz M and Klein-Bauernschmitt P (1999) Enhanced sensitivity of small cell lung cancer cell lines to cisplatin and etoposide after infection with adeno-associated virus type 2. *Eur J Cancer* **35**: 106–110

Hoerer M, Bodegain C, Scheer U, Heberger C, Streyerer S, Burger A and Maass G (1997) The use of recombinant adeno-associated viral vector for transduction of epithelial tumor cells. *Int J Immunopharmacol* **19**: 473–479

Khleif SN, Myers T, Carter BJ and Trempe JP (1991) Inhibition of cellular transformation by the adeno-associated virus rep gene. *Virology* **181**: 738–741

Kube DM, Ponnazhagan S and Srivastava A (1997) Encapsidation of adeno-associated virus type 2 Rep proteins in wild-type and recombinant progeny virions: Rep-mediated growth inhibition of primary human cells. *J Virol* **71**: 7361–7371

Lui H-S, Jan M-S, Chou C-K, Chen P-H and N-J-Ke (1999) Is green fluorescent protein toxic to the living cells. *Biochem Biophys Res Commun* **260**: 712–717

Maass G, Bodegain C, Scheer U, Michl D, Horer M, Braun-Falco M, Volkenrandt M, Schadendorf D, Wendtner CM, Winnacker EL, Kotin RM and Hallek M (1998) Recombinant Adeno-Associated Virus for the generation of autologous, gene-modified tumor vaccines: evidence for high transduction efficiency into primary epithelial cancer cells. *Hum Gene Ther* **9**: 1049–1059

Mah C, Qing K, Khuntirat B, Ponnazhagan S, Wang XS, Kube DM, Yoder MC and Srivastava A (1998) Adeno-associated virus type 2 mediated gene transfer: role of epidermal growth factor receptor protein tyrosine kinase and gene expression. *J Virol* **72**: 9835–9843

Maxwell IH, Maxwell F and Schaack J (1998) An adenovirus type 5 mutant with the preterminal protein gene deleted efficiently provides helper functions for the production of recombinant adeno-associated virus. *J Virol* **72**: 8371–8372

Peng D, Qian C, Sun Y, Barajas MA and Prieto J (2000) Transduction of hepatocellular carcinoma (HCC) using recombinant adeno-associated virus (rAAV): in vitro and in vivo effects of genotoxic agents. *J Hepatol* **32**: 975–985

Qing K, Wang X-S, Kube DM, Ponnazhagan S, Bajpai A and Srivastava A (1997) Role of the tyrosine phosphorylation of a cellular protein in adeno-associated virus mediated gene transfer. *Proc Natl Acad Sci USA* **94**: 10879–10884

Qing K, Khuntirat B, Mah C, Kube DM, Wang XS, Ponnazgahan S, Zhou S, Dwarki VJ, Yoder MC and Srivavstava (1998) Adeno-associated virus type 2 mediated gene transfer: correlation of tyrosine phophorelation of the cellular single stranded D sequence binding protein with transgene expression in human cells in vitro and murine tissues in vivo. *J Virol* **72**: 1593–1599

Samulski RJ, Chang LS and Shenk T (1989) Helper-free stocks of recombinant adeno-associated viruses: Normal integration does not require viral gene expression. *J Virol* **63**: 3822–3828

Schaack J, Guo X and Langer SJ (1996) Characterization of a replication-incompetent adenovirus type 5 mutant deleted for the preterminal protein gene. *Proc Natl Acad Sci USA* **93**: 14686–14691

Schlehofer JR (1994) The tumor suppressive properties of adeno-associated viruses. *Mutat Res* **305**: 303–313

Takeuchi T, Kozuka T, Nakagawa K, Aoki Y, Ohtomo K, Yoshiike K and Kanda T (2000) Adeno-associated virus type 2 nonstructural protein Rep 78 suppresses translation in vitro. *Virology* **266**: 196–202

Tamayose K, Hirai Y and Shimada T (1996) A new strategy for large-scale preparation of high titer recombinant Adeno-Associated Virus vectors by using packaging cell lines and sulfonated cellulose column chromatography. *Hum Gene Ther* **7**: 507–513

Tenenbaum L, Hamdane M, Pouzet M, Avalosse B, Stathopoulos A, Jurysta F, Rosenbaum C, Hanemann CO, Levivier M and Velu T (1999) Cellular contaminants of adeno-associated virus vector stocks can enhance transduction. *Gene Ther* **6**: 1045–1053

Veldwijk MR, Schiedlmeier B, Kleinschmidt JA, Zeller WJ and Fruehauf S (1999) Superior gene transfer into solid tumor cells than into human mobilized peripheral blood progenitor cells using helpervirus free adeno-associated viral vector stocks. *Eur J Cancer* **35**: 1136–1142

Walz C, Schlehofer JR, Flentje M, Rudat V and zur Hausen H (1992) Adeno-associated virus sensitizes HeLa cell tumors to gamma rays. *J Virol* **66**: 5651–5657

Zolothukin S, Potter M, Hauswirth WW, Guy J and Muzyczka N (1996) A “humanized” green fluorescent protein cDNA adapted for high-level expression in mammalian cells. *J Virol* **70**: 4646–4654

*© 2001 Cancer Research Campaign*
